# An *in‐cell* helicase reporter system for quantifying DDX3X and DDX3Y activities

**DOI:** 10.1002/btm2.10720

**Published:** 2025-04-18

**Authors:** Zhi Sheng Poh, James Chia Wei Tan, Brandon Han Siang Wong, Kottaiswamy Amuthavalli, Holy Kristanti, Suat Hoon Tan, Nicholas Francis Grigoropoulos, Navin Kumar Verma

**Affiliations:** ^1^ Lee Kong Chian School of Medicine Nanyang Technological University Singapore Singapore; ^2^ Interdisciplinary Graduate Programme, NTU Institute for Health Technologies (HealthTech NTU) Nanyang Technological University Singapore Singapore; ^3^ School of Chemistry, Chemical Engineering and Biotechnology Nanyang Technological University Singapore Singapore; ^4^ National Skin Centre Singapore; ^5^ Department of Haematology Singapore General Hospital Singapore; ^6^ Duke‐NUS Medical School Singapore; ^7^ Skin Research Institute of Singapore Singapore

**Keywords:** cancer, DDX3X, luciferase, reporter cells, RNA helicase

## Abstract

Genome sequencing has identified numerous mutations in the DEAD‐box RNA helicases, *DDX3X* and *DDX3Y*, associated with cancer and other diseases, but monitoring of their functional consequences remains a challenge. Conventional helicase assays are laborious, often technically difficult, and are performed in cell‐free systems that do not address biologically relevant questions. Here, we developed an engineered DDX3 reporter cell system capable of interrogating helicase activities of DDX3X and DDX3Y and their mutational variants. For this, we deleted the endogenous *DDX3X* in human 293T cells using CRISPR/Cas9. DDX3Y is absent in 293T cells being a female‐derived line. We transfected cells with firefly luciferase plasmids that provided bioluminescence signals, depending on helicase activities of exogenously expressed wild‐type or mutant *DDX3X* or *DDX3Y*, and inserted *Aequorea coerulescens* Green Fluorescent Protein (AcGFP) as an internal control separated by an internal ribosome entry site (IRES). The developed reporter system can be applied to screen compound libraries targeting DDX3X or DDX3Y in living cells and study their functional roles in health and disease.

**Translational Impact Statement**

The novel *in‐cell* DDX3 helicase (ICD‐helicase) reporter system developed in the current study can be used to perform high throughput screening of compound libraries and agents specifically targeting *DDX3X* or *DDX3Y*, implicated in cancer and several other diseases.

## 1 INTRODUCTION

DDX3 is a member of the highly conserved family of DEAD‐box RNA helicases involved in the unwinding of RNA duplexes and mRNA translation.^1^ Humans express two homologues of the DDX3 protein—DDX3X and DDX3Y. The DDX3X protein is encoded by the *DDX3X* gene on p11.3–11.23 of the X‐chromosome and is ubiquitously expressed in all tissues.^2^ The DDX3Y protein is encoded by the paralogous *DDX3Y* gene located in the non‐recombining region of the Y‐chromosome. DDX3Y mRNA is present throughout all the tissues in males, but DDX3Y protein expression is restricted to the testis where it plays an essential role in spermatogenesis and male fertility.^3^

Both DDX3X and DDX3Y share ~92% similarity in amino acid sequences and play an indispensable and often compensatory roles in various cellular processes, including pre‐mRNA splicing, mRNA stability and translation, as well as in signal transduction such as the Wnt/β‐catenin and NF‐κB pathways.^4^ In addition, they regulate a wide range of biological processes, including cell adhesion, cell cycle, and cellular stress responses.^4^, ^5^, ^6^ Germline inheritance of pathogenic DDX3X mutations hinders neurodevelopment, accounting for ~1%–3% cases with intellectual disability.^7^ In cancer, *DDX3X* can function as a tumor suppressor or as an oncogene depending on tumor type.^4^ For example, DDX3X is overexpressed in breast, colorectal, lung, medulloblastoma, and prostate cancers,^4^, ^8^ whereas it is somatically mutated in medulloblastoma, melanoma, and non‐Hodgkin lymphoma subtypes, such as natural killer/T‐cell lymphoma (NKTCL), Burkitt's lymphoma (BL), and diffuse large B‐cell lymphoma (DLBCL).^4^, ^9^, ^10^, ^11^, ^12^

In comparison to DDX3X, biological roles of DDX3Y are relatively less well understood. This could be mainly due to stage‐specific expression of DDX3Y in the male testis during spermatogenesis that makes this protein challenging to isolate and characterize its molecular function. Nevertheless, due to their diverse and crucial roles in cell biology and multiple diseases, both DDX3X and DDX3Y are gaining increasing attention for research.

Although conventional helicase and ATPase assays have been utilized to determine the functions of DDX3X and DDX3Y and identify their pathogenic variants, these assays are complicated and time‐consuming, and often technically challenging due to the requirement of protein synthesis and radiolabelling. Moreover, conventional assays are performed in cell‐free systems which cannot address biologically relevant questions involving multiple cell signaling and molecular factors. To circumvent these challenges, we report a novel engineered cell‐based reporter system for evaluating helicase activities of DDX3X and DDX3Y. The reporter system, herein referred to as *in‐cell* DDX3 helicase (ICD‐helicase) assay, relies on exogenously expressed DDX3X or DDX3Y coupled with firefly *luciferase* in cultured mammalian cells.

## 2 EXPERIMENTAL

### 2.1 Cell culture

Human 293T cells (ATCC #CRL‐3216) were cultured in Dulbecco's modified Eagle's medium (DMEM) supplemented with 10% heat‐inactivated fetal bovine serum (HI‐FBS). DDX3X WT and DDX3X R475C mutant U2932 DLBCL cells were cultured in Roswell Park Memorial Institute (RPMI) 1640 medium supplemented with 10% HI‐FBS. Method for generating the DDX3X mutant U2932 cells has been described previously.^9^ Cells were maintained at 37°C in 5% CO_2_ incubator. Cells underwent routine mycoplasma testing with MycoGuard™ Mycoplasma PCR Detection Kit (Genecopoeia #MP004) and were verified free of mycoplasma prior to the commencement of experiments.

### 2.2 Cell transfection

The 293T cells were seeded in 12‐well plate (1.5 × 10^5^ cells per well) a day prior to transfection. Cells were transfected with 1 μg plasmids using Lipofectamine 2000 (ThermoFisher Scientific #11668019) as per manufacturer's recommendations.

### 2.3 CRISPR‐Cas9 knockout

The plasmid expressing Cas9 from *Streptococcus pyogenes* with 2A‐EGFP and a cloning backbone for the guide sgRNA [pSpCas9(BB)‐2A‐GFP (PX458), a gift from Feng Zhang (Addgene plasmid # 48138; http://n2t.net/addgene:48138; RRID:Addgene_48138)]^13^ was used to create stable deletion of *DDX3X* in 293T cells. The sgRNAs target sequences (5′ → 3′) were: DDX3X sg1 (CGTGGACGGAGTGATTACGA), DDX3X sg2 (CGGAGTGATTACGATGGCAT), HBEGF sg10 (GCAAATATGTGAAGGAGCTC), and Cas9 control (GTGTAGTTCGACCATTCGTG). For the enrichment of the *DDX3X* knockout (KO) cell population, we selected *DDX3X*‐deleted 293T cells using diphtheria toxin (DT, Sigma #D0564). Briefly, 293T cells were co‐transfected with plasmids expressing sgRNA against DDX3X and the heparin‐binding EGF‐like growth factor (HBEGF).^14^ Subsequently, cells were treated with 20 ng/mL DT that depleted all the non‐KO cells. Surviving single cell clones were selected to establish the *DDX3X* KO cells. KO efficiency was tested by Western immunoblotting and further confirmed using PCR against genomic DNA at the targeted region.

### 2.4 Plasmids

All expression plasmids for transient expression were cloned into pLVX vector backbone using standard restriction cloning. Site‐directed mutagenesis was performed using Q5® Site‐Directed Mutagenesis Kit (NEB #E0554) to generate pathogenic DDX3X mutants. Sanger sequencing was performed to confirm successful mutation at the desired site. The reporter construct used in our study was made by Vectorbuilder with vector ID VB220922‐1486nnk (pcDNA3.1(+)‐5′‐UTR_RAC1:luciferase) and VB220922‐1483jcb (pcDNA3.1(+)‐5′‐UTR_DVL2:luciferase). The 5′UTR‐luciferase was subsequently subcloned into pIRES2‐AcGFP1 (TakaraBio #632435) using standard restriction cloning with *Nhe* and *Xho*I restriction sites. pcDNA3.1+ eiF4A1 myc HIS (Addgene plasmid # 71657; http://n2t.net/addgene:71657; RRID:Addgene_71657) and pcDNA3.1(+) eiF4A2 myc HIS (Addgene plasmid # 71658; http://n2t.net/addgene:71658; RRID:Addgene_71658) were gifts from Gideon Dreyfuss. pDESTmycDDX17 was a gift from Thomas Tuschl (Addgene plasmid # 19876; http://n2t.net/addgene:19876; RRID:Addgene_19876).^15^ p23‐DDX21 WT was a gift from Ling‐Ling Chen (Addgene plasmid # 128803; http://n2t.net/addgene:128803; RRID:Addgene_128803).^16^ pFRT‐DHX36iso1 was a gift from Markus Hafner (Addgene plasmid # 159585; http://n2t.net/addgene:159585; RRID:Addgene_159585).^17^

### 2.5 Luciferase assay

After experimental treatments, cells were detached from culture plates. Half of the cells were lysed in luciferase cell lysis buffer (Promega #E1500) before measuring bioluminescence in a white bottom 96‐well plate using luciferase assay system (Promega #E4030) as per manufacturer's instructions. The other half of the cells were subjected to flow‐cytometry to determine the percentage of AcGFP^+^ cells. Normalized luciferase activity was first determined by normalizing AcGFP^+^ cells within each experiment against sgCTL population to account for variation in transfection efficiency. Cycloheximide (Sigma #01810) was used as a positive control for translation inhibition at 1 μg/mL and RK‐33 (Selleck #S8246) was used as a positive control for chemical inhibition of DDX3 at various concentrations as indicated in respective figure legends. All the inhibitors were dissolved in dimethyl sulfoxide (DMSO) to a final concentration of 0.1% (v/v) and subsequently added to the cell culture medium.

### 2.6 Puromycin incorporation assay

Prior to pulsing the cells with puromycin, positive control well was pre‐treated with cycloheximide (1 μg/mL) for 3 h to inhibit translation. Subsequently, all the wells were subjected to puromycin (Gibco #A1113803) at 1 μg/mL for 30 min except for negative control well before harvesting of cell lysates. Global protein synthesis was determined by normalizing puromycin incorporation bands against ponceau S staining.

### 2.7 Western immunoblotting

Cells were washed with ice‐cold phosphate buffer saline and subsequently lysed to extract cellular proteins, as described earlier.^9^ Extracted proteins were quantified via Bradford Assay (Bio‐Rad #5000001) and equal amounts of cell lysates (20–40 μg) from various samples were resolved on 8%–20% gradient gel. Proteins were then transferred from the gel onto an activated PVDF membrane using Trans‐Blot Turbo Transfer System (Bio‐Rad #1704156). Membrane was incubated in 3% skim milk in tris buffer saline 0.1% Tween‐20 (TBS‐T) for 30 min at room temperature before incubating with relevant primary antibodies overnight at 4°C with gentle shaking. The following primary antibodies were used: anti‐DDX3X (Bethyl Laboratories #A300‐474A), anti‐GAPDH (Millipore #AB2302), anti‐Flag (Sigma #F1804), anti‐HA (Merck #11867423001), anti‐Myc (Invitrogen #MA1‐21316), and anti‐Puromycin (Sigma #MABE343). Primary antibodies were used at a concentration around 250 ng/mL. The next day, membranes were washed three times in TBS‐T before incubating with the appropriate HRP secondary antibodies. For secondary antibodies, anti‐rabbit HRP conjugate (Jackson Immunoresearch #111‐035‐144) or anti‐mouse HRP conjugate (Jackson Immunoresearch #115‐035‐166) were used as when appropriate (1:7000 dilution). Blots were visualized on the ChemiDoc Imaging Systems (Bio‐Rad) using SuperSignal™ West Pico PLUS Chemiluminescent Substrate (ThermoFisher Scientific #34580), Clarity Western ECL Substrate (Bio‐Rad #1705061), or Clarity Max Western ECL Substrate (Bio‐Rad #1705062) when appropriate. The uncropped blots are provided in Appendix S1.

### 2.8 Data analysis

Statistical analysis was performed as described in the figure legends with either *t*‐tests (for comparison between two groups), one‐way ANOVA (for comparison between >2 experimental groups), or two‐way ANOVA (multiple comparison within experimental groups) using GraphPad Prism 9.3.0 software. *p* values <0.05 were considered statistically significant.

## 3 RESULTS AND DISCUSSION

### 3.1 The ICD‐helicase reporter cell system accurately measures DDX3 helicase function

**FIGURE 1:**
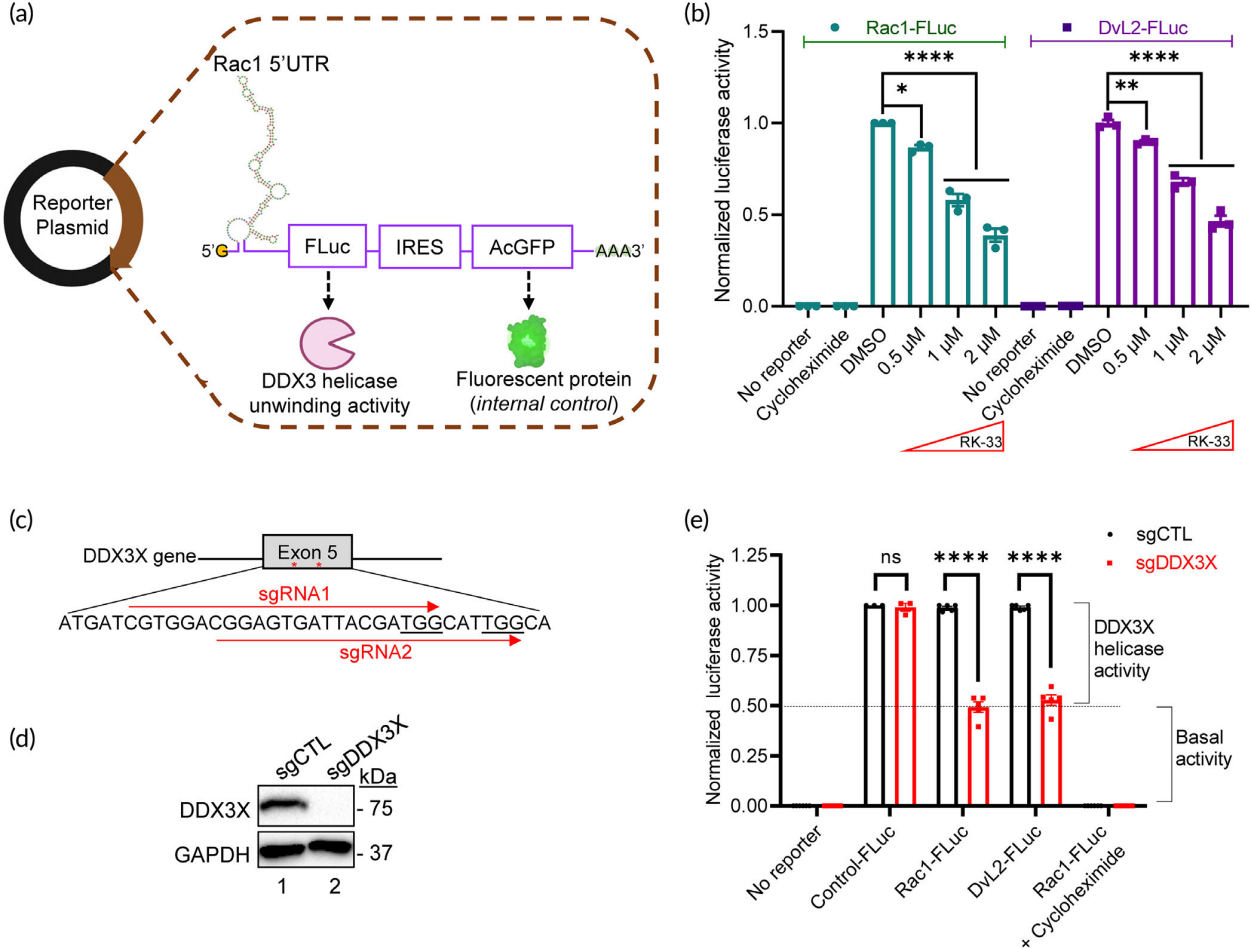
Design and development of the novel ICD‐helicase reporter cell system. (a) Schematic of reporter plasmid in which the complex Rac1 5′UTR was placed upstream of the firefly *luciferase* (*FLuc*). The *Aequorea coerulescens* green fluorescence protein (AcGFP) was placed downstream as an internal control separated by an internal ribosome entry site (IRES). RNA secondary structure was created using ViennaRNA. (b) The 293T cells transfected with *Rac1‐FLuc* or *DvL2‐FLuc* reporter plasmids emitting luciferase signals in the presence of vehicle (DMSO) that were dose‐dependently inhibited by RK‐33 in the concentration range of 0.5–2 μM. Cells were treated with cycloheximide as an inhibitory control. (c) Schematic and sequences of CRISPR/Cas9 KO target sites for two different gRNAs against the *DDX3X* gene (*sgRNA1* and *sgRNA2*). (d) Representative immunoblots of control (*sgCTL*) and DDX3X KO (*sgDDX3X*) 293T cells probed for DDX3X and GAPDH (*protein loading control*). (e) Relative luciferase activities in WT (*sgCTL*) and DDX3X KO (*sgDDX3X*) 293T *Rac1‐FLuc* and *DvL2‐FLuc* reporter cells. Firefly luciferase activity was determined by normalizing AcGFP^+^ levels within each sample before comparing against sgCTL firefly luciferase signals. Cells were treated with cycloheximide as an inhibitory control. Data represent three independent experiments and values in the bars are mean ± SEM. *p < 0.05; **p < 0.01; ****p < 0.0001; ns, non‐significant.

The helicase function of the DDX3X and DDX3Y proteins regulates mRNA translation by unwinding of the complex 5′ untranslated region (5′UTR) secondary structure.^18^ We selected two unique 5′UTR structures—(1) Rac1 5′UTR and (2) DvL2 5′UTR that are regulated by DDX3X and DDX3Y. The 5′UTR segments were placed upstream of the *luciferase* gene (*Fluc*, from the firefly *Photinus pyralis*) driven by a constitutively active human cytomegalovirus promoter along with an *Aequorea coerulescens* Green Fluorescent Protein (AcGFP) as an internal control separated by an *internal ribosome entry site* (IRES) (Figures 1a and S1a). Since luciferase emits bioluminescence signals proportional to its levels of expression, it served as a readout for helicase unwinding activity in ICD‐helicase assays while AcGFP signals were used to normalize for variation in transfection efficiency (Figure S1b). We then transiently transfected reporter plasmids (Rac1‐FLuc or DvL2‐FLuc) into 293T cells. Our choice for the selection of this cell line was for three main reasons. First, the 293T cell line originates from a female and does not express the Y‐chromosome homolog DDX3Y. Second, 293T cells can tolerate KO or nearly complete knock‐down of DDX3X expression.^19^ Third, it is relatively easy to re‐transduce WT or mutant plasmids in the KO/transgenic 293T cells. Treatment of 293T cells expressing Rac1‐FLuc or DvL2‐FLuc bicistronic reporter with a non‐specific DDX3 inhibitor RK‐33,^20^ in the concentrations range of 0.5–2 μM, resulted in a dose‐dependent inhibition of luciferase signals (Figure 1b). This concentration range was chosen because ≥3 μM RK‐33 has been reported to be toxic to various cell lines in culture.^21^, ^22^ RK‐33 concentrations up to 2 μM were tolerable to cells (Figure S1c).

Since endogenous DDX3X in 293T cells can interfere with exogenously expressed DDX3X or DDX3Y, we knocked‐out *DDX3X* in these cells using CRISPR/Cas9 (*sgDDX3X*; Figure 1c). Complete depletion of DDX3X protein in sgDDX3X 293T cells was confirmed by Western immunoblotting (Figure 1d) and these cells remained viable in culture (Figure S1d,e). DDX3X‐depleted sgDDX3X 293T cells exhibited significantly reduced luciferase signals in both Rac1‐FLuc and DvL2‐FLuc reporters with respective 5′UTRs, but not in the cells without 5′UTR (Control‐Fluc; Figure 1e). Similar results were obtained with the four other isogenic sgDDX3X clones that we generated (Figure S2a,b). Moreover, in the absence of the DDX3 protein, DDX3X KO 293T cells expressing either Rac1‐FLuc or DvL2‐FLuc construct did not respond to RK‐33 (Figure S2c). Notably, DDX3X‐depleted reporter cells exhibited a basal level of luciferase signals. This could be due to other members of the DEAD box helicase family proteins present in sgDDX3X 293T cells that can also partially unwind the complex 5′UTR. To investigate this possibility, we exogenously expressed five closely‐related DEAD‐box helicases *eIF4A1*, *eIF4A2*, *DDX5*, *DDX21*, and *DDX17* as well as the DEAH‐box helicase *DHX36* [selected based on the similarity with DDX3^23^] in sgDDX3X reporter cells (Figure S3a). We noted increased luciferase signals in both Rac1‐FLuc and DvL2‐FLuc 5′UTRs due to either of the six DEAD/DEAH‐box helicases (Figure S3b,c). Collectively, these results established Rac1‐FLuc and DvL2‐Fluc reporters to be sensitive to DDX3X alteration.

### 3.2 Cancer associated DDX3X mutants have dysfunctional helicase activity

**FIGURE 2:**
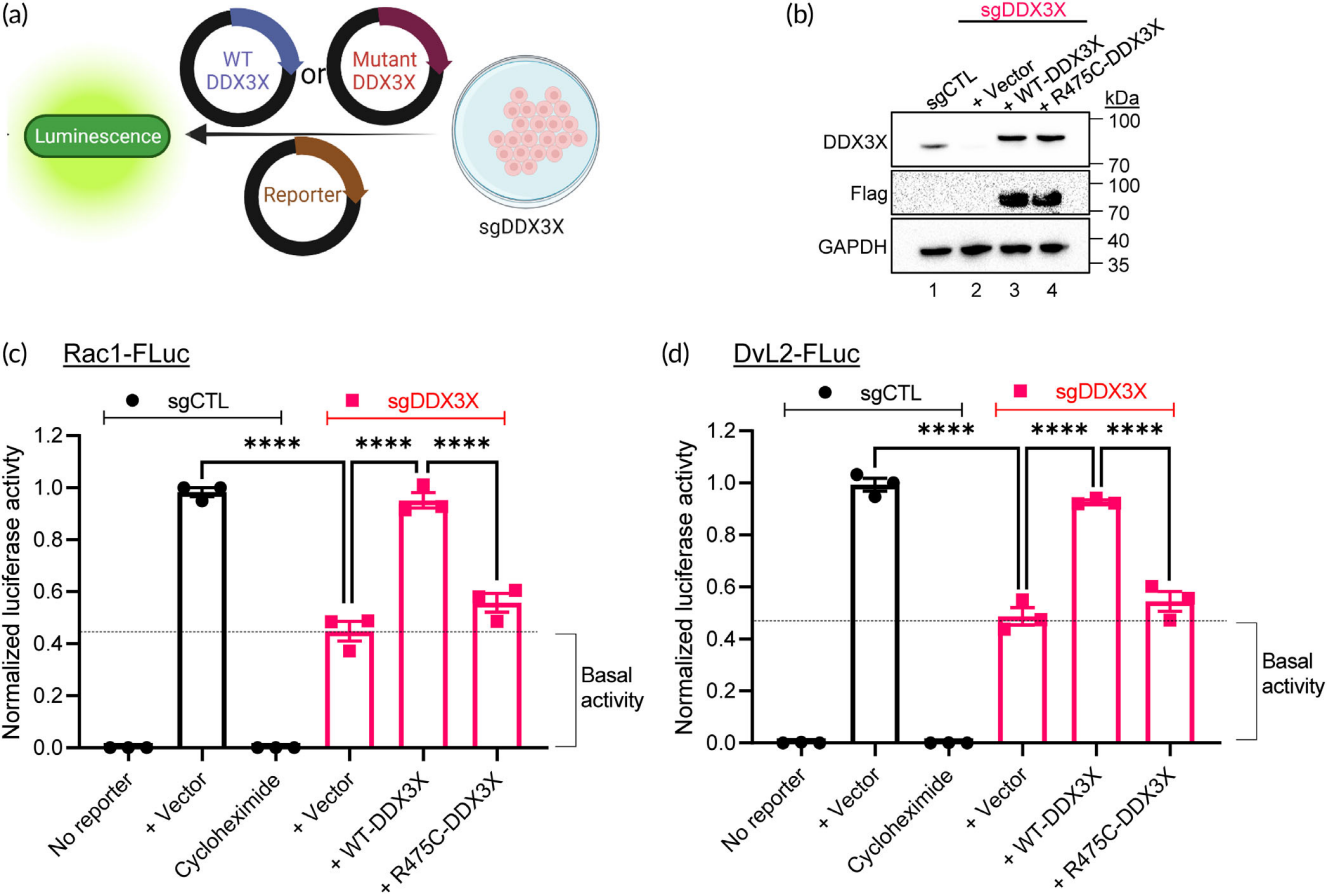
Determination of helicase activities of WT and mutant *DDX3X*. (a) Schematic for interrogating helicase activities of WT and mutant *DDX3X*. (b) Representative immunoblots of sgCTL and sgDDX3X 293T cells transfected with WT‐ or R475‐DDX3X plasmid constructs, probed for DDX3X, Flag and GAPDH (loading control). Densitometry quantification of the DDX3X blot is provided as Appendix S1, Figure S4a. (c, d) Control (*sgCTL*) and DDX3X KO (*sgDDX3X*) 293T cells were transfected with plasmid vector alone, WT‐DDX3X, or R475C‐DDX3X plasmids. Relative luciferase activities of reporter cells with (c) Rac1‐FLuc or (d) DvL2‐FLuc were determined by normalizing AcGFP^+^ levels within each sample before comparing against sgCTL firefly luciferase signals. Cells were treated with cycloheximide as an inhibitory control. Data represent three independent experiments and values in the bars are mean ± SEM. ****p < 0.0001.

We have earlier identified nine different mutations in the *DDX3X* gene in a cohort of relapsed/refractory DLBCL patients.^9^ Specifically, the mutation at the amino acid residue 475 that resides in the RNA binding domain (motif IVa)^24^ of *DDX3X* (*R475C‐DDX3X*) has been found to be a recurring mutation in many non‐Hodgkin lymphoma subtypes,^10^, ^12^, ^25^ and has been associated with aggressiveness and chemoresistance in DLBCL.^9^ We therefore choose to model *R475C‐DDX3X* mutation in the current study and determined the helicase activity of this mutant compared to WT‐*DDX3X*. For this, we exogeneously expressed *WT‐DDX3X* or mutant *R475C‐DDX3X* plasmids in sgDDX3X 293T cells (Figure 2a) and confirmed their expression by Western immunoblotting (Figures 2b and S4a). Reconstituting sgDDX3X 293T cells with *WT‐DDX3X* rescued the ability of cells to unwind the target 5′UTRs, while expression of *R475C‐DDX3X* failed to unwind the target 5′UTRs (Figure 2c,d), demonstrating loss of helicase activity of the mutant R475C‐DDX3X protein.

### 3.3 DDX3X somatic mutants identified in a subgroup of lymphoma patients exhibit loss of helicase function

**FIGURE 3:**
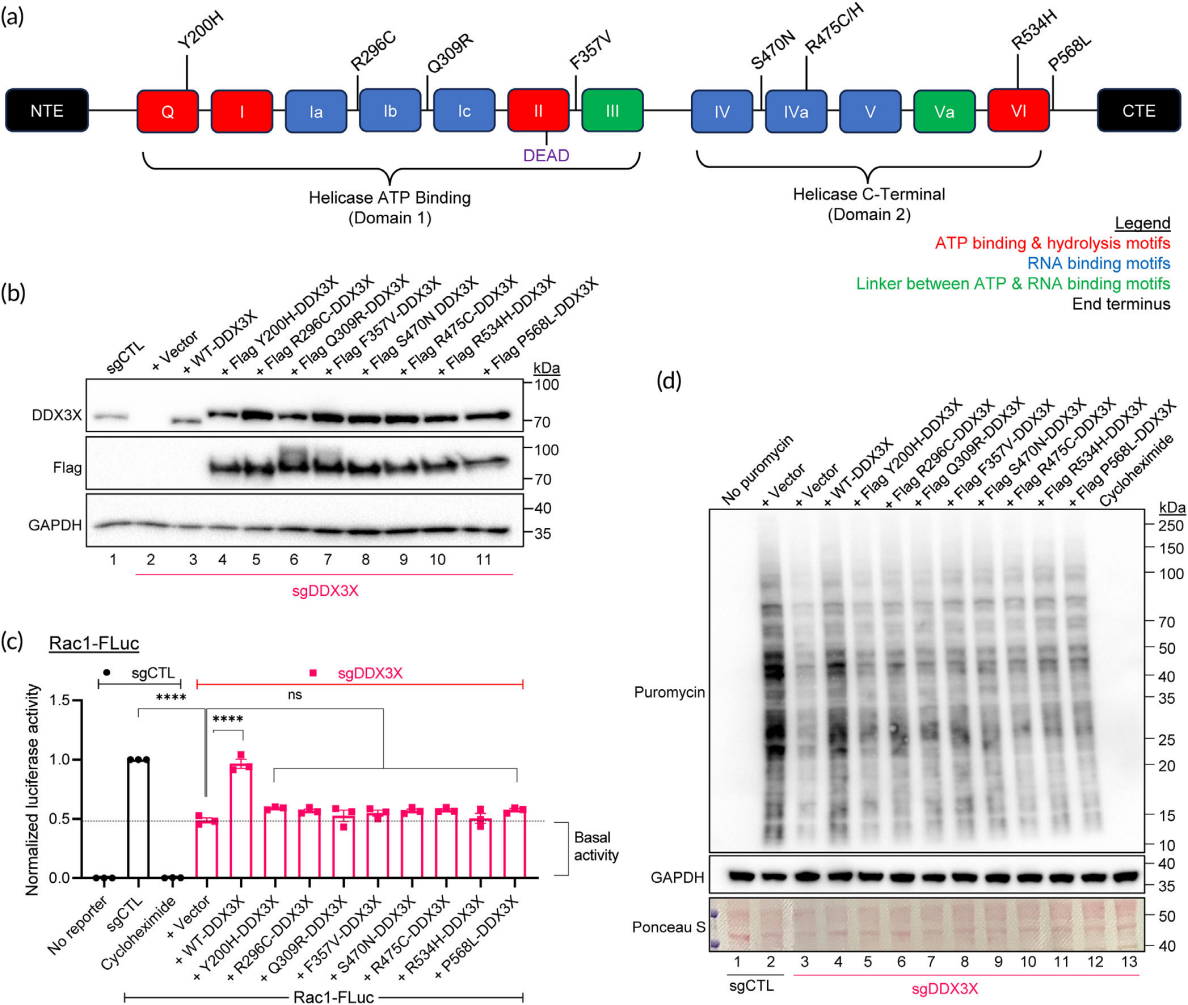
Interrogation of the helicase activities of mutant DDX3X identified in DLBCL patients. (a) An illustration of mutational landscape of *DDX3X* in DLBCL patients based on previously reported studies.^4^, ^9^ The 12 helicase motifs of *DDX3X* that are involved in ATP binding and hydrolysis, RNA binding, and linker between RNA and ATP binding sites are shown. CTE, C‐terminal extension; NTE, N‐terminal extension. (b) Representative immunoblots of control (*sgCTL*) and DDX3X KO (*sgDDX3X*) 293T cells transfected with Flag‐tagged DDX3X WT or mutant plasmids as indicated. Blot was probed for DDX3X and Flag to confirm exogenous expression of mutant DDX3X and GAPDH as a protein loading control. Densitometry quantification of the DDX3X blot is provided as Appendix S1 and Figure S4b. (c) Relative luciferase activities of reporter cells produced by WT or mutant DDX3X as indicated, determined by normalizing AcGFP^+^ levels within each sample before comparing against sgCTL firefly luciferase signals. Cells were treated with cycloheximide as a translation inhibitory control. (d) sgCTL and sgDDX3X 293T cells transfected with Flag‐tagged WT or mutant DDX3X plasmids as indicated were subjected to puromycin incorporation assay, lysed and cellular lysates were analyzed by Western immunoblotting. The blot was also probed for GAPDH and stained with Ponceau S to confirm equal protein loading. Data represent three independent experiments and values in the bars are mean ± SEM. ****p < 0.0001; ns, non‐significant.

Of the nine mutations that we have earlier reported in DLBCL patients,^9^ four (Y200H, R475C/H, and R534H) are located in the conserved motifs Q, Iva, and VI, and the remaining five (R296C, Q309R, F357V, S470N, and P568L) are dispersed within the conserved helicase core of domains 1 and 2 (Figure 3a). PolyPhen‐2 bioinformatic analysis predicted these mutations to be “damaging.”^9^ To confirm whether these mutations cause loss of helicase activity in cells, we generated plasmid vectors containing eight of the above *DDX3X* point mutations and expressed them in the sgDDX3X Rac1‐FLuc 293T reporter cells (Figure 3b, lanes 4–11). Luciferase assays showed that none of the eight *DDX3X* mutants could unwind the target 5′UTR mRNA as effectively as WT‐*DDX3X* (Figure 3c).

Since DDX3X is involved in the pre‐assembly of translation–initiation complexes to facilitate cap‐dependent translation via its helicase activity,^26^ we subjected all the above transgenic cells to a puromycin incorporation assay that measures global protein synthesis. Results showed that protein synthesis in cells expressing the *DDX3X* mutant plasmids was significantly impaired (Figure 3d, lanes 5–11). Since all the eight *DDX3X* point‐mutations have been found to be associated with poor prognosis in DLBCL patients,^9^, ^27^ it can be suggested that inability of DDX3X to unwind RNA plays either a direct or an indirect role in DLBCL progression and/or chemoresistance.

**FIGURE 4:**
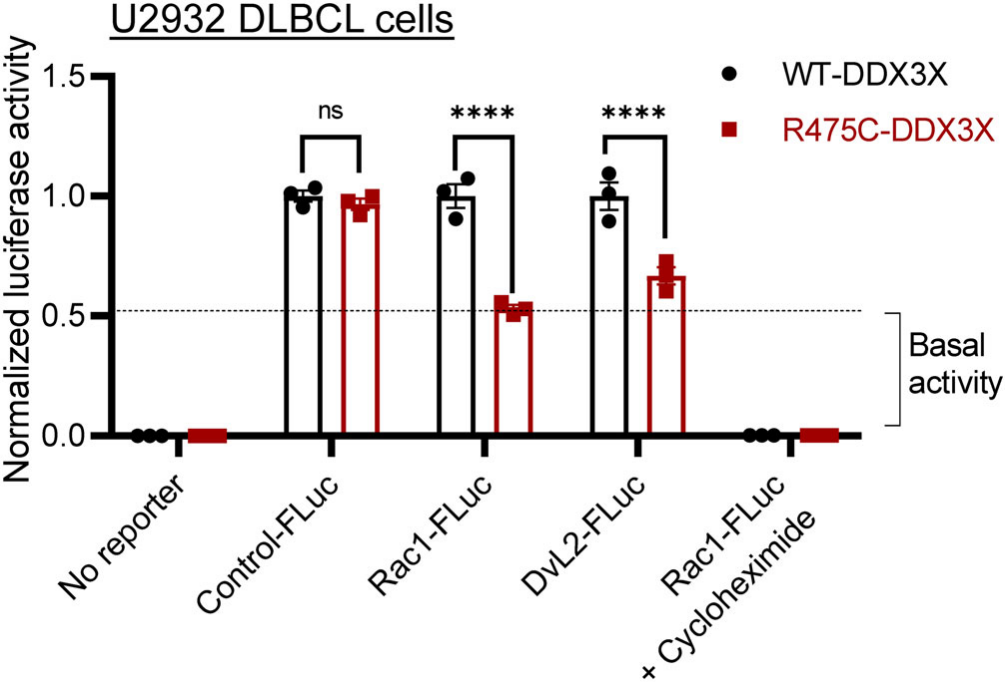
Quantification of DDX3X helicase activity in DLBCL cells. Relative luciferase signals of Rac1‐FLuc and DvL2‐FLuc U2932 reporter cells expressing WT‐DDX3X or mutant R475C‐DDX3X determined by normalizing AcGFP^+^ levels within each sample. Cells were treated with cycloheximide as a translation inhibitory control. Values are mean ± SEM of three independent experiments. ****p < 0.001; ns, non‐significant.

To substantiate the helicase‐dead phenotype of mutant DDX3X in non‐Hodgkin lymphoma patients, we extended the *DDX3X* gene engineering approach to the U2932 cell line that was established from ascites in a patient suffering from DLBCL.^28^ For this, we first generated a point mutation at the R475C residue in the *DDX3X* gene (*DDX3X*‐*R475C*) in U2932 cells using the CRISPR/Cas9 knock‐in technique, as described.^25^ Subsequently, reporter plasmids were expressed in wild‐type and R475C mutant U2932 cells and luciferase signals were recorded. In comparison to U2932 cells with WT‐*DDX3X*, cells containing *R475C‐DDX3X* mutation exhibited significantly impaired helicase unwinding activity with Rac1‐FLuc and DvL2‐FLuc (Figure 4).

### 3.4 ICD‐helicase cell systems reveal subtle differences between DDX3X and DDX3Y helicase functions

**FIGURE 5:**
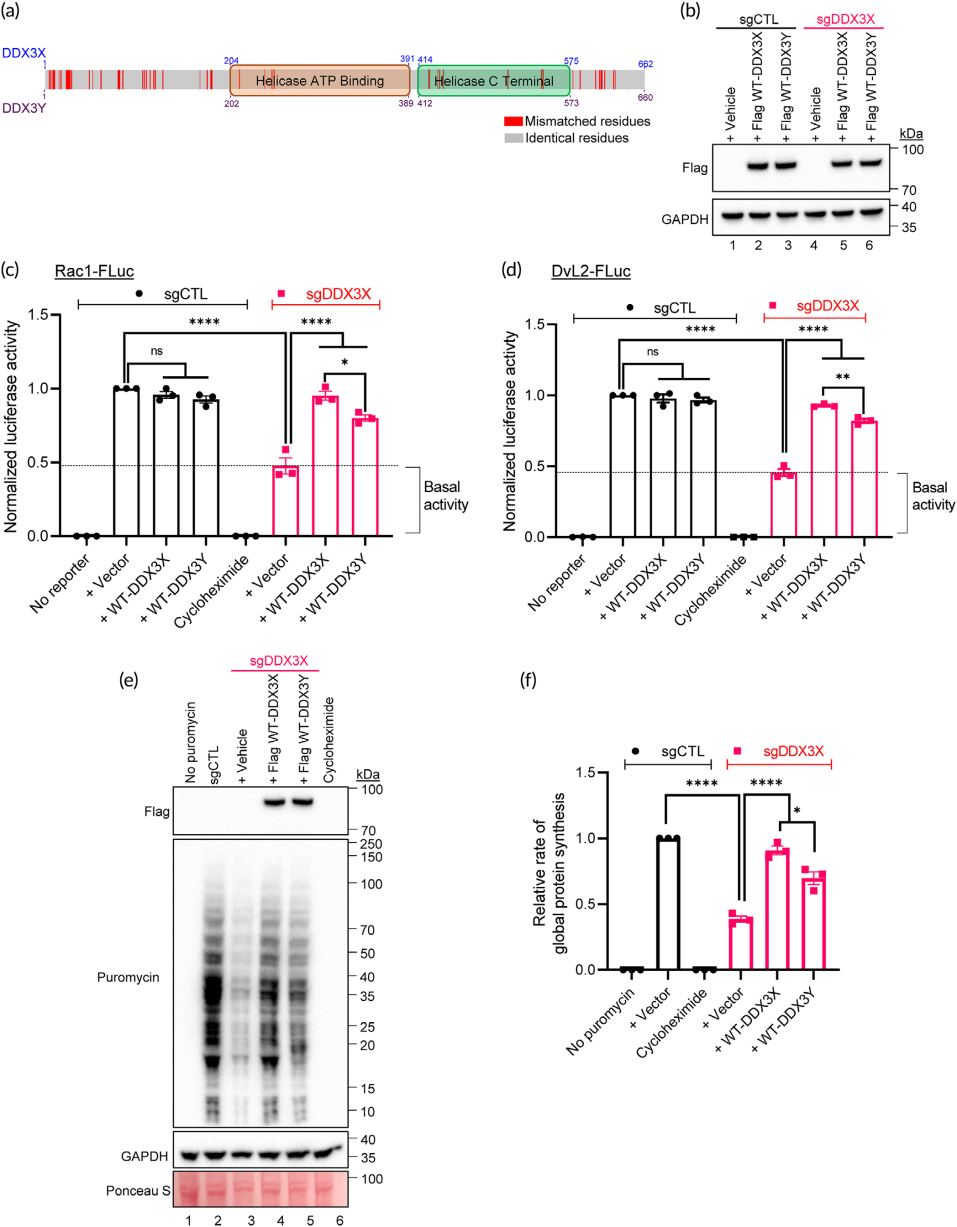
Comparative helicase activities of DDX3X and DDX3Y using the ICD‐helicase system. (a) An illustration of DDX3X and DDX3Y amino acid sequence alignment. Red denotes mismatched residues and gray denotes identical residues. (b) Representative immunoblots of sgCTL and sgDDX3X 293T cells transfected with Flag‐DDX3X/Y constructs, probed for Flag (for DDX3X/Y) and GAPDH (loading control). (c, d) Firefly luciferase activities of (c) Rac1‐FLuc and (d) DvL2‐FLuc reporter cells under indicated treatment conditions were determined by normalizing AcGFP^+^ levels within each sample before comparing against sgCTL firefly luciferase signals. Cells were treated with cycloheximide as a translation inhibitory control. (e) sgCTL and sgDDX3X 293T cells transfected with Flag‐tagged WT‐DDX3X or WT‐DDX3Y plasmids were subjected to puromycin incorporation assay, lysed and cellular lysates were analyzed by Western immunoblotting. The blot was also probed for GAPDH and stained with Ponceau S to confirm equal protein loading. (f) Densitometric analysis of puromycin incorporation to determine relative levels of global protein synthesis. Data represent three independent experiments and values in the bars are mean ± SEM. *p < 0.05; **p < 0.01; ****p < 0.0001; ns, non‐significant.

To further define the sensitivity of our developed ICD‐helicase reporter system, we extended the usage of this tool to interrogate the poorly characterized Y‐chromosome homolog, DDX3Y. DDX3X and DDX3Y share functional homology,^29^, ^30^ with intrinsically disordered regions (IDR) in each of the N and C termini of the non‐helicase regions (Figure 5a). Sequence alignment (Figure S5a) revealed a conserved nucleotide binding domain necessary for helicase activity indicating that DDX3Y may interact with ATP and double stranded RNA in a manner similar to DDX3X. Since DDX3Y can compensate for the loss of DDX3X function,^25^ we expressed WT *DDX3X* or WT *DDX3Y* in our 293T reporter cell system to confirm this observation (Figure 5b). In control sgCTL cells, overexpression of either WT *DDX3X* or WT *DDX3Y* did not cause any significant change in the luminescence signal in both Rac1‐FLuc and DvL2‐FLuc 5′UTRs (Figure 5c,d; black bars), probably due to a saturation in DDX3 helicase unwinding activities by endogenous proteins. Exogenous expression of WT *DDX3X* or WT *DDX3Y* in sgDDX3X 293T cells (cells without endogenous DDX3X and DDX3Y) could rescue the helicase unwinding activity of cells (Figure 5c,d; red bars). Similarly, exogenous expression of WT *DDX3X* or WT *DDX3Y* in sgDDX3X 293T cells could rescue the ability of cells to translate proteins (Figure 5e, lanes 4 and 5 vs. lane 3). We also noted that DDX3Y helicase is less efficient in RNA unwinding as well as protein translation compared to DDX3X (Figure 5e,f), suggesting subtle functional differences despite both proteins sharing >90% sequence homologies.

We next extended the utility of the reporter cell system to quantify mouse Ddx3 (mDdx3) helicase activity. In contrast to the two homologous genes (*DDX3X* and *DDX3Y*) present in humans, there are three homologues DDX3 genes (*mDdx3x*, *mDdx3y*, and *D1Pas1–PL10*) in mice.^31^, ^32^ Both mouse Ddx3x and Ddx3y are ubiquitously expressed throughout the mouse with >99% homology with human DDX3X and DDX3Y, suggesting conserved function between species.^5^ However, *D1Pas1–PL10* is expressed only in mouse sperm.^5^ Exogenous expression of mouse *WT‐Ddx3x* or *WT‐Ddx3y* (Figure S3b) in sgDDX3X 293T cells could rescue the helicase unwinding activity of cells similar to the levels of human WT‐DDX3X (Figure S3c). Collectively, these data demonstrate the versatility of our reporter system beyond human DDX3 paralogs.

### 3.5 Helicase and ATPase activities are necessary for DDX3Y function

**FIGURE 6:**
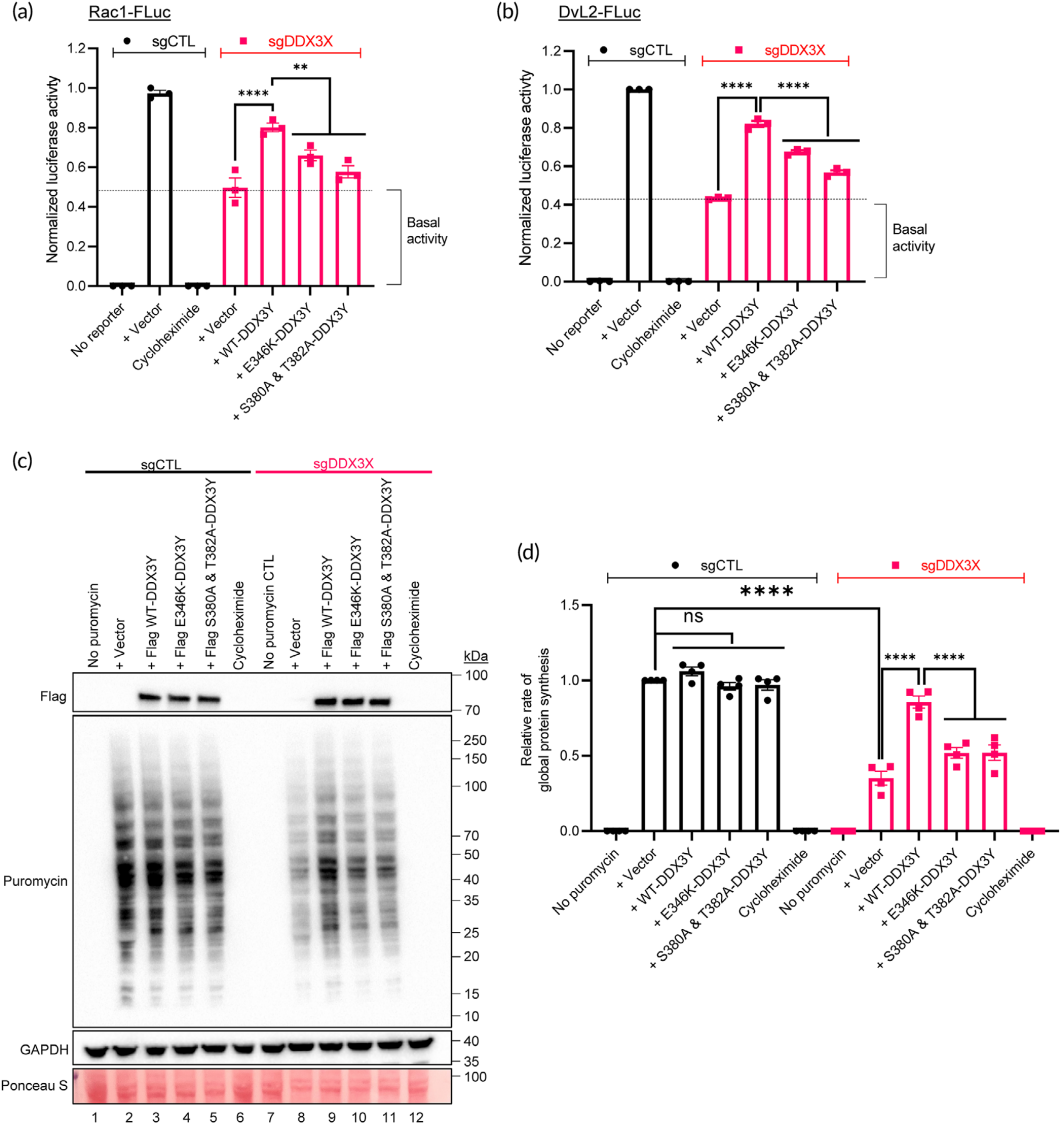
Both helicase and ATPase activities are necessary for DDX3Y function. Relative luciferase signals produced by WT or mutant DDX3Y as indicated in sgCTL and sgDDX3X Rac1‐FLuc (a) and DvL2‐FLuc (b) reporter cells, determined by normalizing AcGFP^+^ levels within each sample before comparing against sgCTL firefly luciferase signals. Cells were treated with cycloheximide as an inhibitory control. (c) sgCTL and sgDDX3X 293T cells transfected with WT or mutant DDX3Y plasmids as indicated were subjected to puromycin incorporation assay, lysed and cellular lysates were analyzed by Western immunoblotting. The blot was also probed for GAPDH and stained with Ponceau S to confirm equal protein loading. (d) Densitometric analysis of puromycin incorporation to determine relative levels of global protein synthesis. Data represent three independent experiments and values in the bars are mean ± SEM. **p < 0.01; ****p < 0.0001; ns, non‐significant.

Developing specific inhibitors against either of the DDX3 homologues (DDX3X or DDX3Y) requires an *in depth* understanding of their homolog‐specific functions. Based on sequence homologies of DDX3X and DDX3Y, we generated two defective mutants—(1) *DDX3Y‐E346K* harboring a point‐mutation in the catalytic DEAD‐box domain that impairs its helicase and ATPase activity and (2) *DDX3Y‐S380A* and *T382A* harboring two point‐mutations exhibiting defective helicase activity but retaining the ATPase activity of the helicase. We expressed both mutants in the DDX3 reporter 293T cells and found that both mutations impaired the ability of DDX3Y to rescue cellular luciferase activity in Rac1‐FLuc and DvL2‐FLuc (Figure 6a,b). Similar results were obtained in terms of their inability to rescue global protein synthesis (Figure 6c, lanes 10 and 11 vs. lane 9). A modest increase in protein synthesis as compared to control vector (Figure 6d) indicates that the non‐helicase domains of DDX3Y may be involved in translation regulation through direct or indirect interaction with mRNAs. These suggest that, for designing DDX3Y specific inhibitors, one must consider targeting beyond the helicase region to fully disrupt DDX3Y functions.

## 4 CONCLUSION

In summary, we developed a novel ICD‐helicase reporter cell system that allowed us to determine the helicase activity of DDX3X and DDX3Y. This cell‐based system, in contrast to the conventional approach of performing helicase unwinding assay or ATPase activity assay, offers several advantages. First, the ICD‐helicase reporter cell system enables a quick and accurate screening of multiple pathogenic *DDX3X* and *DDX3Y* mutants. Second, this reporter system does not require recombinant proteins, saving time and resources in obtaining purified proteins. Finally, our approach is cell‐based and provides a more accurate representation for cellular helicase unwinding activity of DDX3X and DDX3Y. Collectively, results highlight the potential of the developed ICD‐helicase reporter cell system in studying the biology of DDX3 in health and DDX3 mutation‐associated diseases.

## AUTHOR CONTRIBUTIONS

**Zhi Sheng Poh:** Conceptualization; methodology; data curation; investigation; validation; formal analysis; writing – original draft. **James Chia Wei Tan:** Methodology; formal analysis; visualization; data curation; investigation; validation. **Brandon Han Siang Wong:** Methodology; investigation; validation; data curation. **Kottaiswamy Amuthavalli:** Methodology; investigation; validation. **Holy Kristanti:** Methodology; investigation; validation. **Suat Hoon Tan:** Conceptualization; supervision; writing – review and editing; formal analysis. **Nicholas Francis Grigoropoulos:** Conceptualization; investigation; writing – review and editing; supervision; funding acquisition; formal analysis. **Navin Kumar Verma:** Conceptualization; investigation; project administration; supervision; writing – review and editing; writing – original draft; funding acquisition; data curation; formal analysis.

## CONFLICT OF INTEREST STATEMENT

The authors (Z. S. P., N. F. G., and N. K. V.) have submitted a Technology Disclosure (NTU Ref: 2023‐388) to the NTUitive Singapore.

## Supporting information


**FIGURE S1:** (a) Schematic of reporter plasmid in which the complex DvL2 5′UTR was placed upstream of the firefly luciferase (FLuc). The *Aequorea coerulescens* green fluorescence protein (AcGFP) was placed downstream as an internal control separated by an internal ribosome entry site (IRES). RNA secondary structure was created using ViennaRNA. (b) Gating strategy adopted to determine transfection efficiency within each sample for normalizing bioluminescence signals. (c) The viability of reporter cells (sgCTL and sgDDX3X) treated with various RK‐33 concentrations (0.5, 1, or 2 μM) was determined by an MTS‐based assay normalized against vehicle (DMSO). Staurosporine (STS) was used as a positive kill control. (d) Representative brightfield images of wild‐type (WT) and engineered sgCTL and sgDDX3X 293T cells. (e) Apoptosis/cell death in control and DDX3X knock‐out 293T cells was determined using an Annexin V‐FITC assay kit. Cells were treated with STS as a kill control. Bar chart represents mean ± SEM of three independent experiments.
**FIGURE S2:** (a) Representative immunoblot of WT, sgCTL, and sgDDX3X 293T cells from various clones showing the expression levels of DDX3X and GAPDH (loading control). (b) Firefly luciferase activity of reporter cells, normalized against AcGFP+ levels within each sample before comparing against sgCTL firefly luciferase activity. Cycloheximide was used as a positive control for translational inhibition. (c) Firefly luciferase activity of sgCTL and sgDDX3X reporter cells (Rac1‐FLuc and DvL2‐FLuc) after treatment with increasing concentrations of RK‐33 (0.5, 1, or 2 μM). Data represent mean ± SEM of at least three independent experiments. ns, non‐significant, *p < 0.05; ****p < 0.0001.
**FIGURE S3:** (a) Representative immunoblots of protein samples obtained from sgDDX3X cells transfected with empty vector or plasmids for Myc‐eIF4A1, Myc‐eIF4A2, HA‐DDX5, Flag‐DDX21, Flag‐DHX36, or Myc‐DDX17 and probed for Myc, HA, or Flag. Blots were re‐probed for GAPDH as a loading control. (b) Firefly luciferase activity of sgCTL and sgDDX3X Rac1‐Fluc as well as DvL2‐FLuc reporter 293T cells expressing eIF4A1 or elF4A2. (c) Firefly luciferase activity of control (sgCTL) and sgDDX3X Rac1‐FLuc as well as DvL2‐FLuc reporter 293T cells expressing DDX5, DDX317, DDX21, or DHX36. Firefly luciferase activity was normalized using AcGFP+ levels within each sample before comparing against sgCTL firefly luciferase activity. Cycloheximide was used as a positive control for translational inhibition. Data represent mean ± SEM of three independent experiments. *p < 0.05; **p < 0.01; ****p < 0.0001.
**FIGURE S4:** (a) Relative densitometry quantification of Western blot bands in protein samples from sgDDX3X cells that were transfected with plasmids to express WT‐DDX3X or R475‐DDX3X are presented as Figure 2b. (b) Relative densitometry quantification of Western blot bands in protein samples from sgDDX3X cells that were transfected with plasmids to express WT‐DDX3X, Y200H, R296C, Q309R, F357V, S470N, R475, R534H, or P568L are presented as Figure 3b. Data represent mean ± SEM of three independent experiments.
**FIGURE S5:** (a) Amino acid sequence alignment between human DDX3X and DDX3Y. Matched residues are in gray while mismatched residues are in red. Nucleotide binding domains are highlighted in pink. (b) Representative immunoblot of sgDDX3X cells transfected with empty vector, mDdx3x or mDdx3y, probed for DDX3 and GAPDH (loading control). (c) Firefly luciferase activity of sgCTL and sgDDX3X 293T cells expressing human DDX3X or mouse mDdx3, normalized using AcGFP+ levels within each sample before comparing against sgCTL firefly luciferase activity. Cycloheximide was used as a positive control for translational inhibition. Data represent mean ± SEM of three independent experiments. ****p < 0.0001.

## Data Availability

The data that support the findings of this study are available from the corresponding author upon reasonable request.
