# Supplementary material for: An in‐cell helicase reporter system for quantifying DDX3X and DDX3Y activities
Source: Bioeng Transl Med. 2025 Apr 18;10(3):e10720. doi: 10.1002/btm2.10720 (PMC12079504; doi:10.1002/btm2.10720)

Supplementary Data

An *In-Cell* Helicase Reporter System for Quantifying DDX3X and DDX3Y Activities

Zhi Sheng Poh[1], James Tan Chia Wei[1] , Brandon Han Siang Wong[1], Kottaiswamy Amuthavalli[1], Holy Kristanti[1], Suat Hoon Tan[2], Nicholas Francis Grigoropoulos[3,4*], and Navin Kumar Verma[1,2,5*]

[1]Lee Kong Chian School of Medicine, Nanyang Technological University Singapore, Singapore

[2]National Skin Centre Singapore, Singapore

[3]Department of Haematology, Singapore General Hospital, Singapore

[4]Duke-NUS Medical School, Singapore

[5]Skin Research Institute of Singapore, Singapore

[*]**Correspondence:** Navin Kumar Verma, Lee Kong Chian School of Medicine, Nanyang Technological University Singapore, 11 Mandalay Road, Clinical Sciences Building, Singapore 308232, Email: nkverma@ntu.edu.sg; Nicholas Francis Grigoropoulos, Department of Haematology, Singapore General Hospital, Singapore 169608, Email: nick.grig@duke-nus.edu.sg

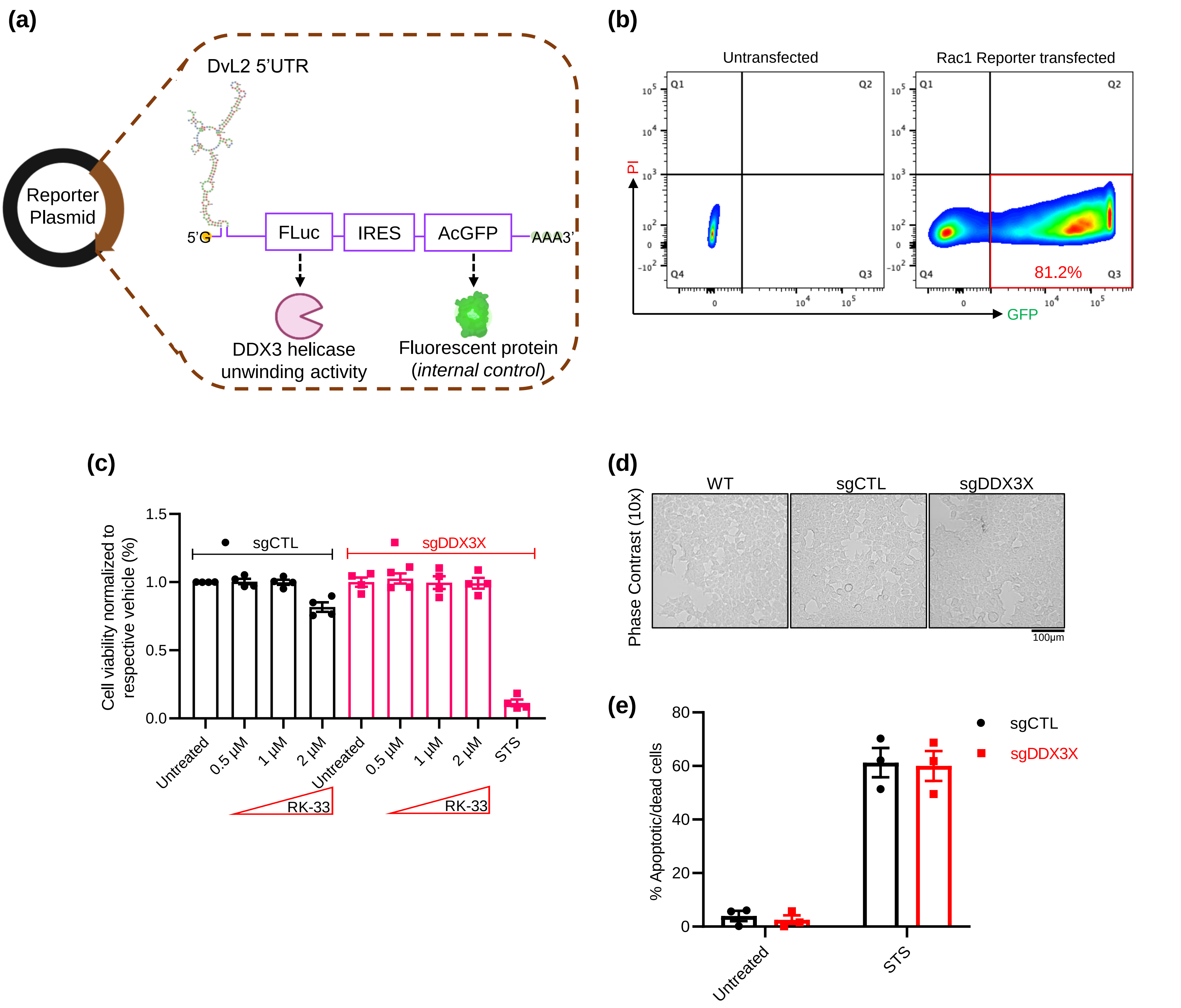

Figure S1. (a) Schematic of reporter plasmid in which the complex DvL2 5'UTR was placed upstream of the firefly luciferase (*FLuc*). The *Aequorea coerulescens* green fluorescence protein (*AcGFP*) was placed downstream as an internal control separated by an *internal ribosome entry site* (*IRES*). RNA secondary structure was created using ViennaRNA. **(b)** Gating strategy adopted to determine transfection efficiency within each sample for normalizing bioluminescence signals. **(c)** The viability of reporter cells (sgCTL and sgDDX3X) treated with various RK-33 concentrations (0.5, 1, or 2 μM) was determined by an MTS-based assay normalized against vehicle (DMSO). Staurosporine (STS) was used as a positive kill control. **(d)** Representative brightfield images of wild-type (*WT*) and engineered sgCTL and sgDDX3X 293T cells. **(e)** Apoptosis/cell death in control and DDX3X knock-out 293T cells was determined using an Annexin V-FITC assay kit. Cells were treated with STS as a kill control. Bar chart represents mean±SEM of 3 independent experiments.

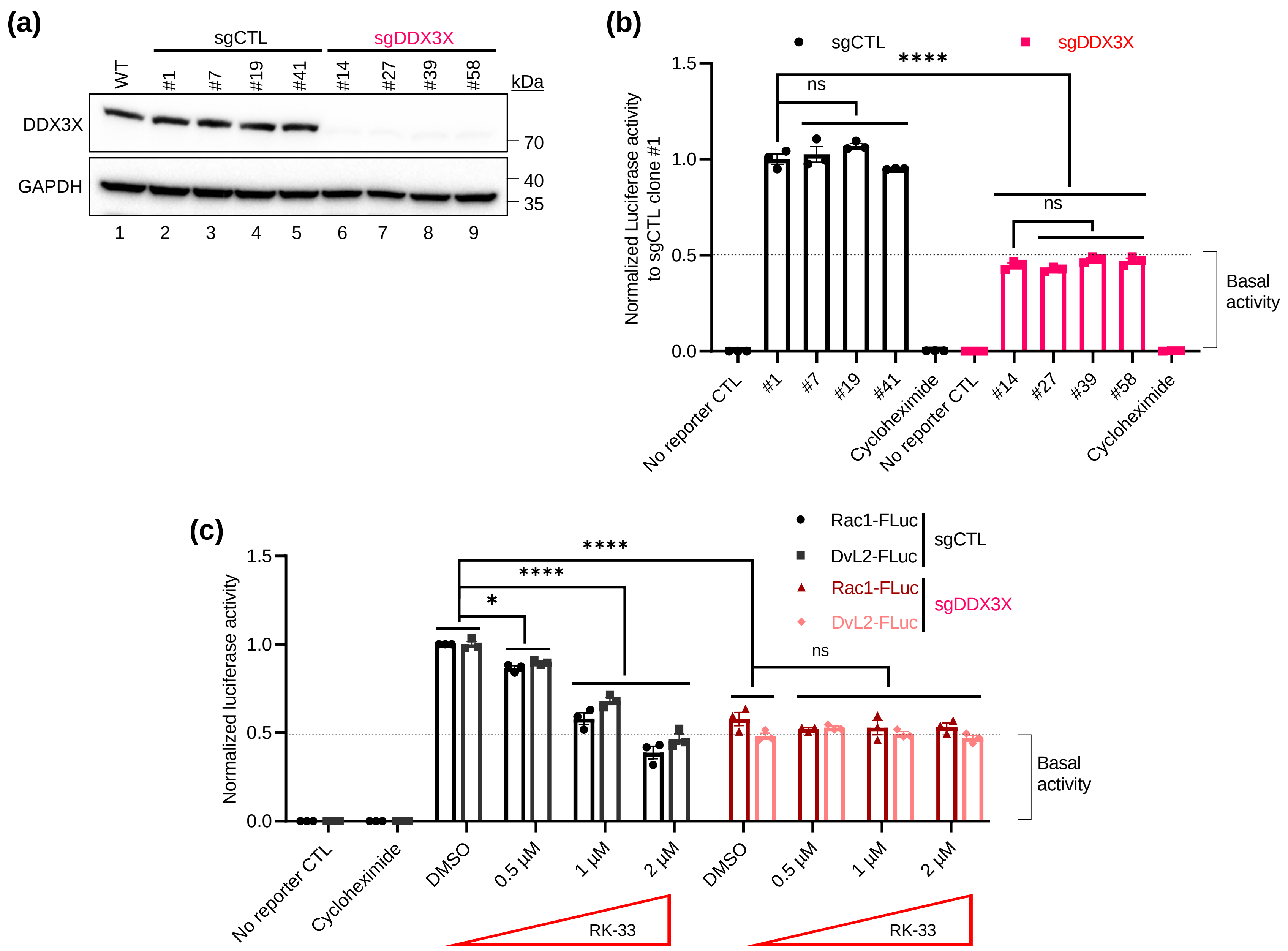

Figure S2. (a) Representative immunoblot of WT, sgCTL and sgDDX3X 293T cells from various clones showing the expression levels of DDX3X and GAPDH (loading control). **(b)** Firefly luciferase activity of reporter cells, normalized against AcGFP^{+} levels within each sample before comparing against sgCTL firefly luciferase activity. Cycloheximide was used as a positive control for translational inhibition. **(c)** Firefly luciferase activity of sgCTL and sgDDX3X reporter cells (Rac1-FLuc & DvL2-FLuc) after treatment with increasing concentrations of RK-33 (0.5, 1, or 2 μM). Data represent mean±SEM of at least 3 independent experiments. ns, non-significant, *, p<0.05; ****, p<0.0001.

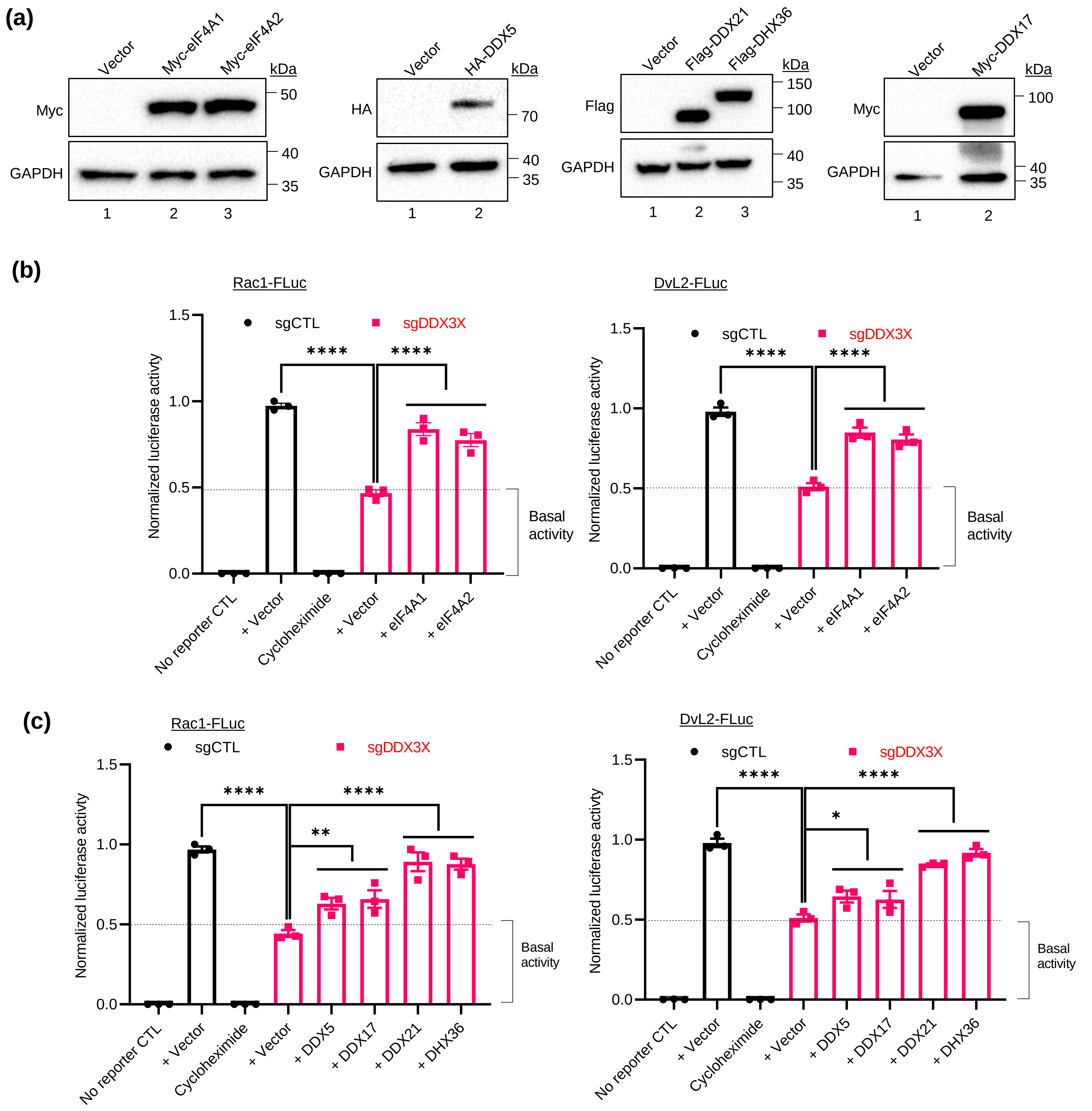

Figure S3. (a) Representative immunoblots of protein samples obtained from sgDDX3X cells transfected with empty vector or plasmids for Myc-eIF4A1, Myc-eIF4A2, HA-DDX5, Flag-DDX21, Flag-DHX36, or Myc-DDX17 and probed for Myc, HA, or Flag. Blots were re-probed for GAPDH as a loading control. **(b)** Firefly luciferase activity of sgCTL and sgDDX3X Rac1-Fluc as well as DvL2-FLuc reporter 293T cells expressing eIF4A1 or eIF4A2. **(c)** Firefly luciferase activity of control (sgCTL) and sgDDX3X Rac1-FLuc as well as DvL2-FLuc reporter 293T cells expressing DDX5, DDX317, DDX21, or DHX36. Firefly luciferase activity was normalized using AcGFP^{+} levels within each sample before comparing against sgCTL firefly luciferase activity. Cycloheximide was used as a positive control for translational inhibition. Data represent mean±SEM of 3 independent experiments. *,p<0.05; **, p<0.01; ****, p<0.0001.

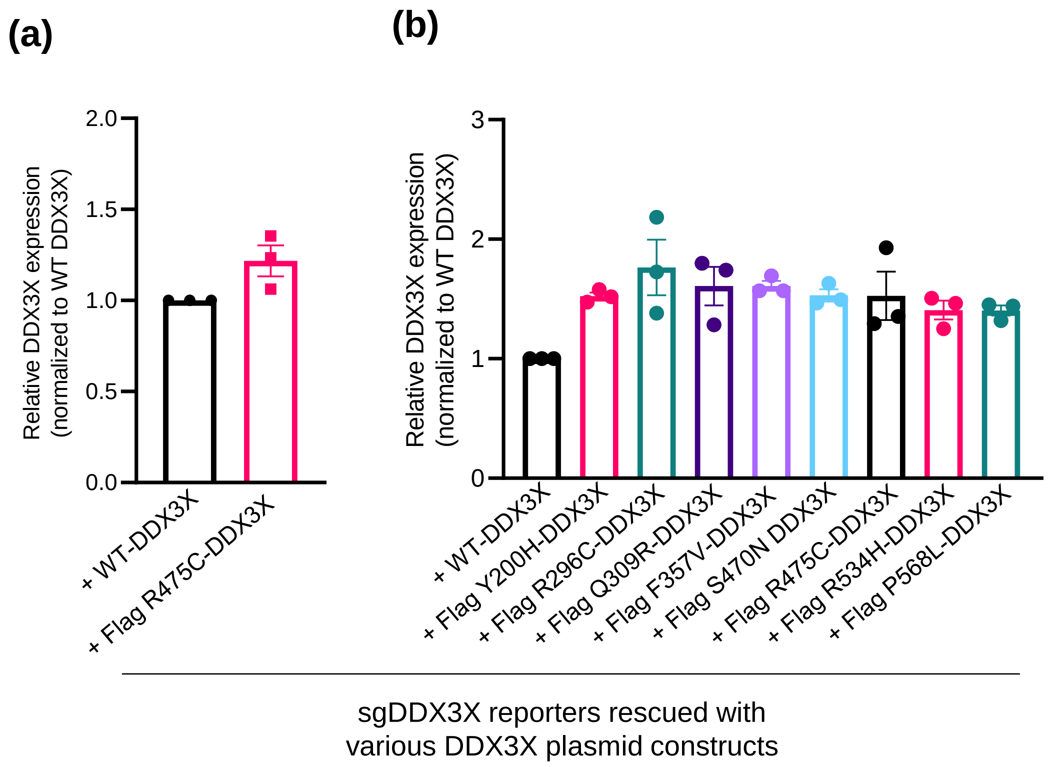

Figure S4. (a) Relative densitometry quantification of Western blot bands in protein samples from sgDDX3X cells that were transfected with plasmids to express WT-DDX3X or R475-DDX3X and presented as the main “Figure 2b”. **(b)** Relative densitometry quantification of Western blot bands in protein samples from sgDDX3X cells that were transfected with plasmids to express WT-DDX3X, Y200H, R296C, Q309R, F357V, S470N, R475, R534H, or P568L and presented as the main “Figure 3b”. Data represent mean ± SEM of 3 independent experiments.

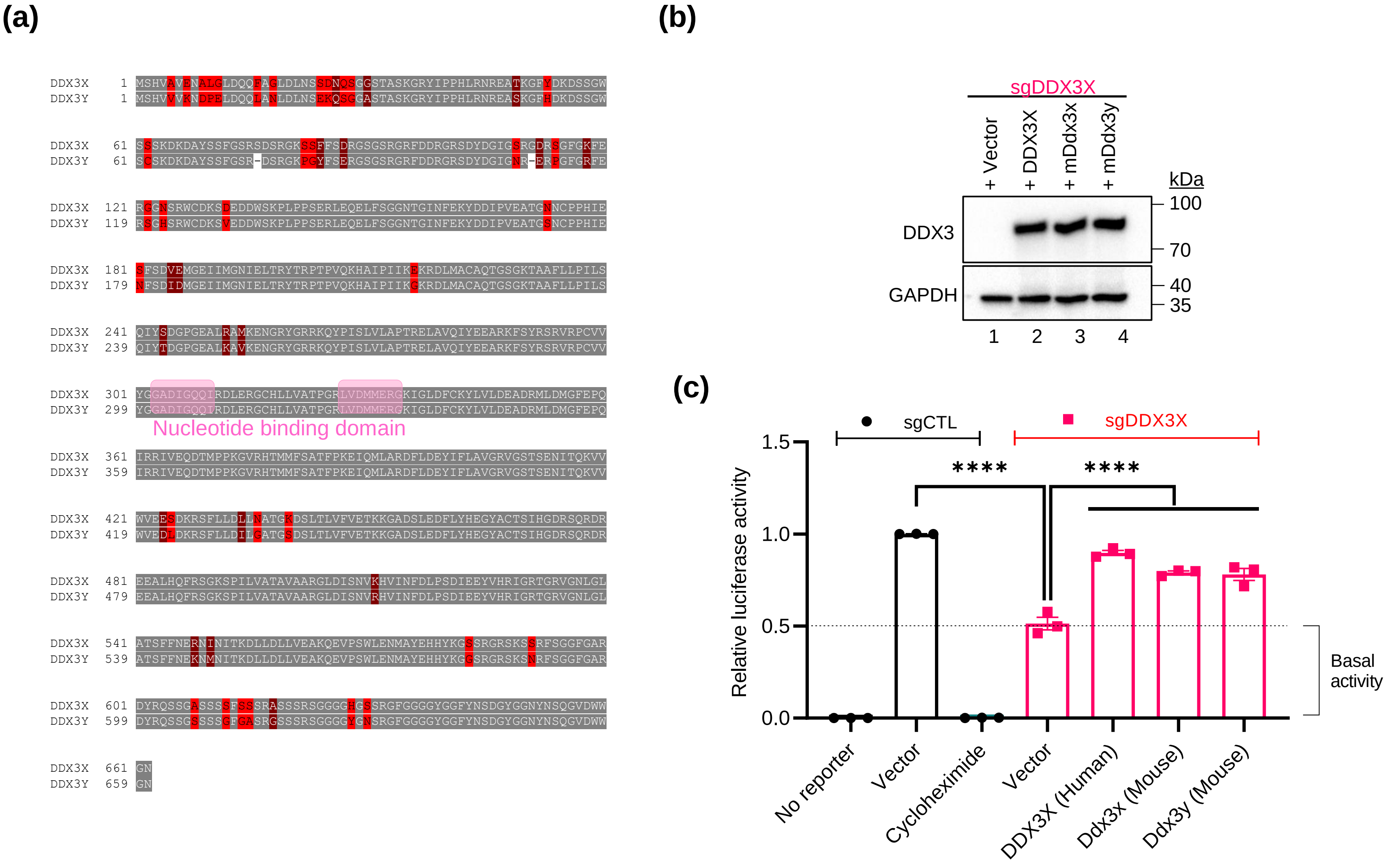

Figure S5. (a) Amino acid sequence alignment between human DDX3X and DDX3Y. Matched residues are in grey while mismatched residues are in red. Nucleotide binding domains are highlighted in pink. **(b)** Representative immunoblot of sgDDX3X cells transfected with empty vector, mDdx3x or mDdx3y, probed for DDX3 and GAPDH (loading control). **(c)** Firefly luciferase activity of sgCTL and sgDDX3X 293T cells expressing human DDX3X or mouse mDdx3, normalized using AcGFP^{+} levels within each sample before comparing against sgCTL firefly luciferase activity. Cycloheximide was used as a positive control for translational inhibition. Data represent mean $\pm$ SEM of 3 independent experiments. ****, $p<0.0001$.

Uncropped blots

Figure 1d

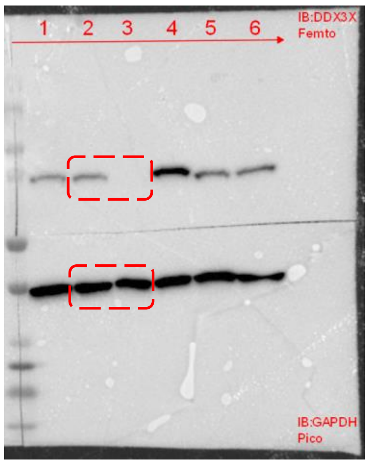

Figure 2b

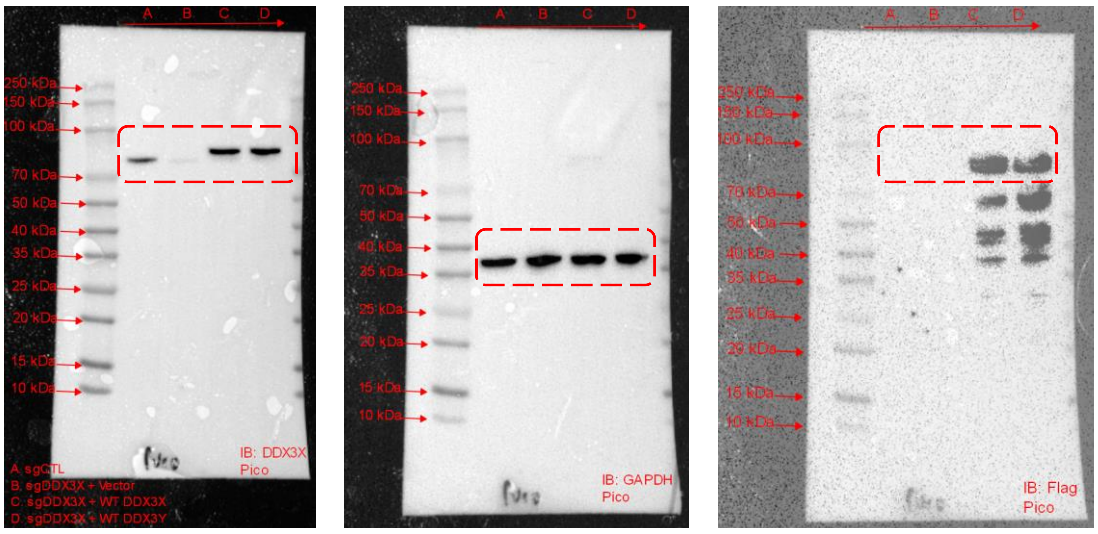

Uncropped blots

Figure 3b

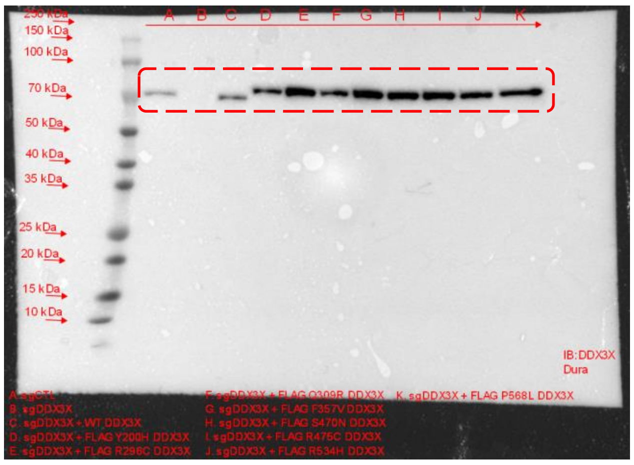

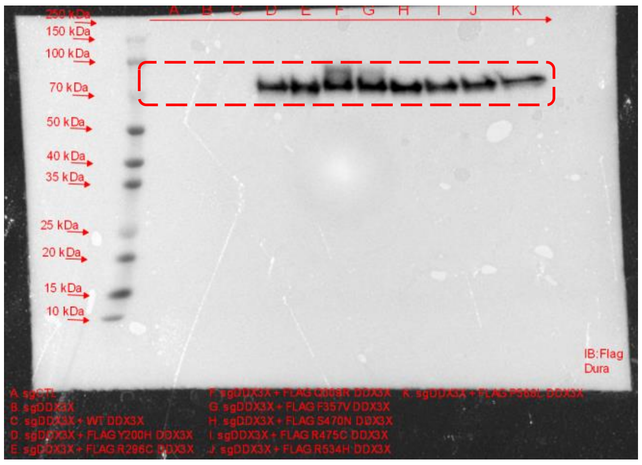

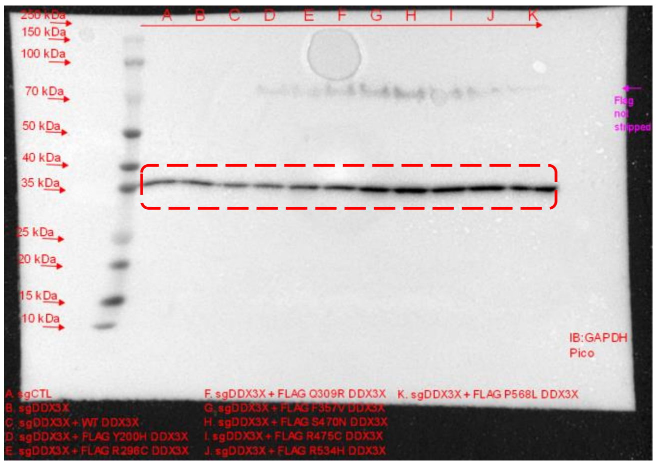

Figure 3d

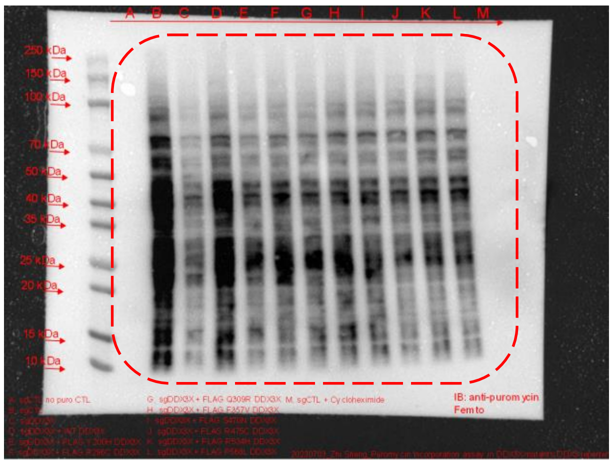

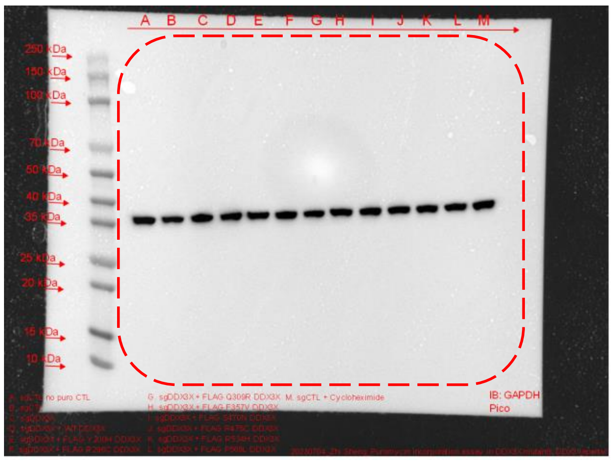

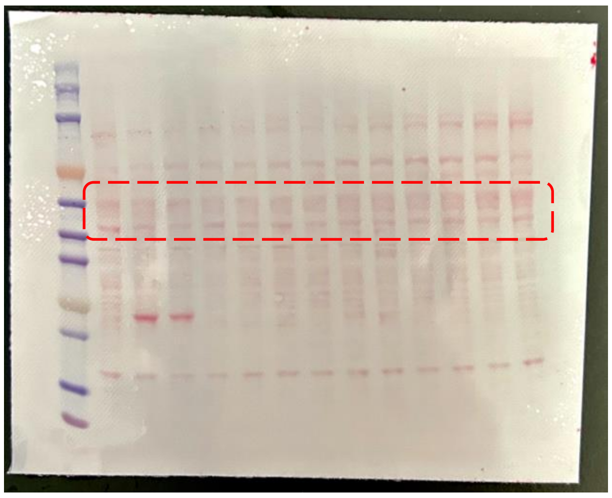

Uncropped blots

Figure 5b

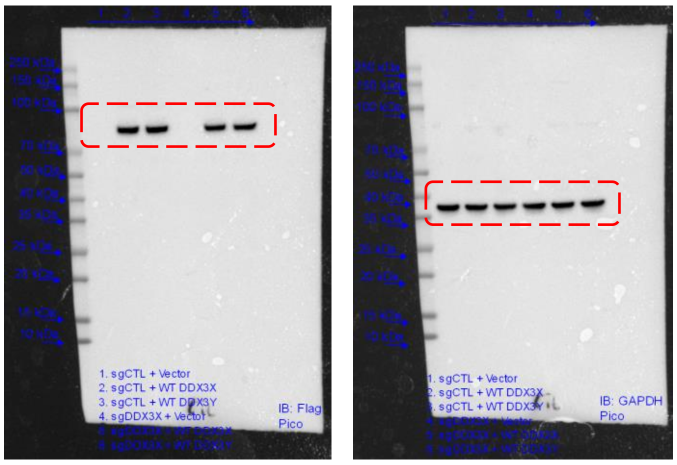

Figure 5e

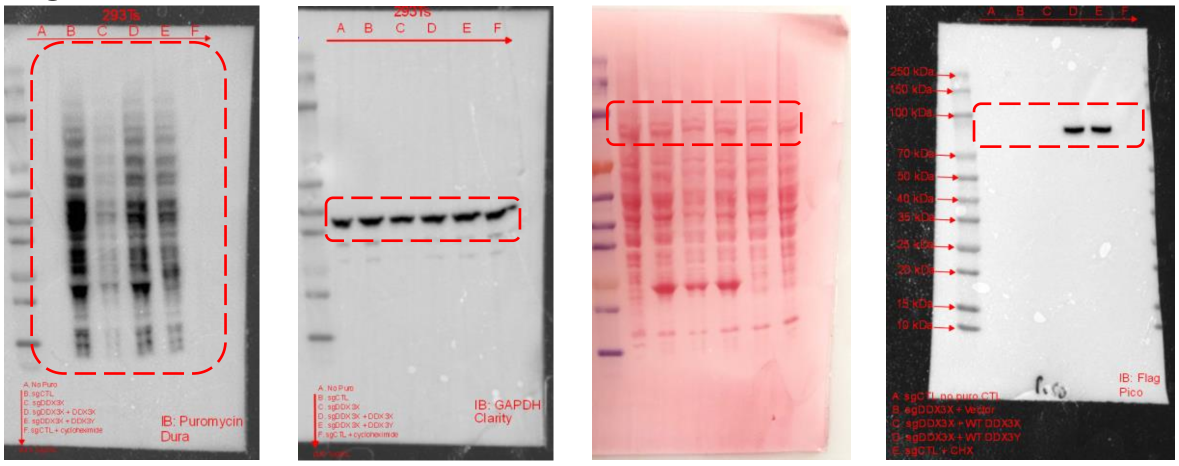

Figure 6c

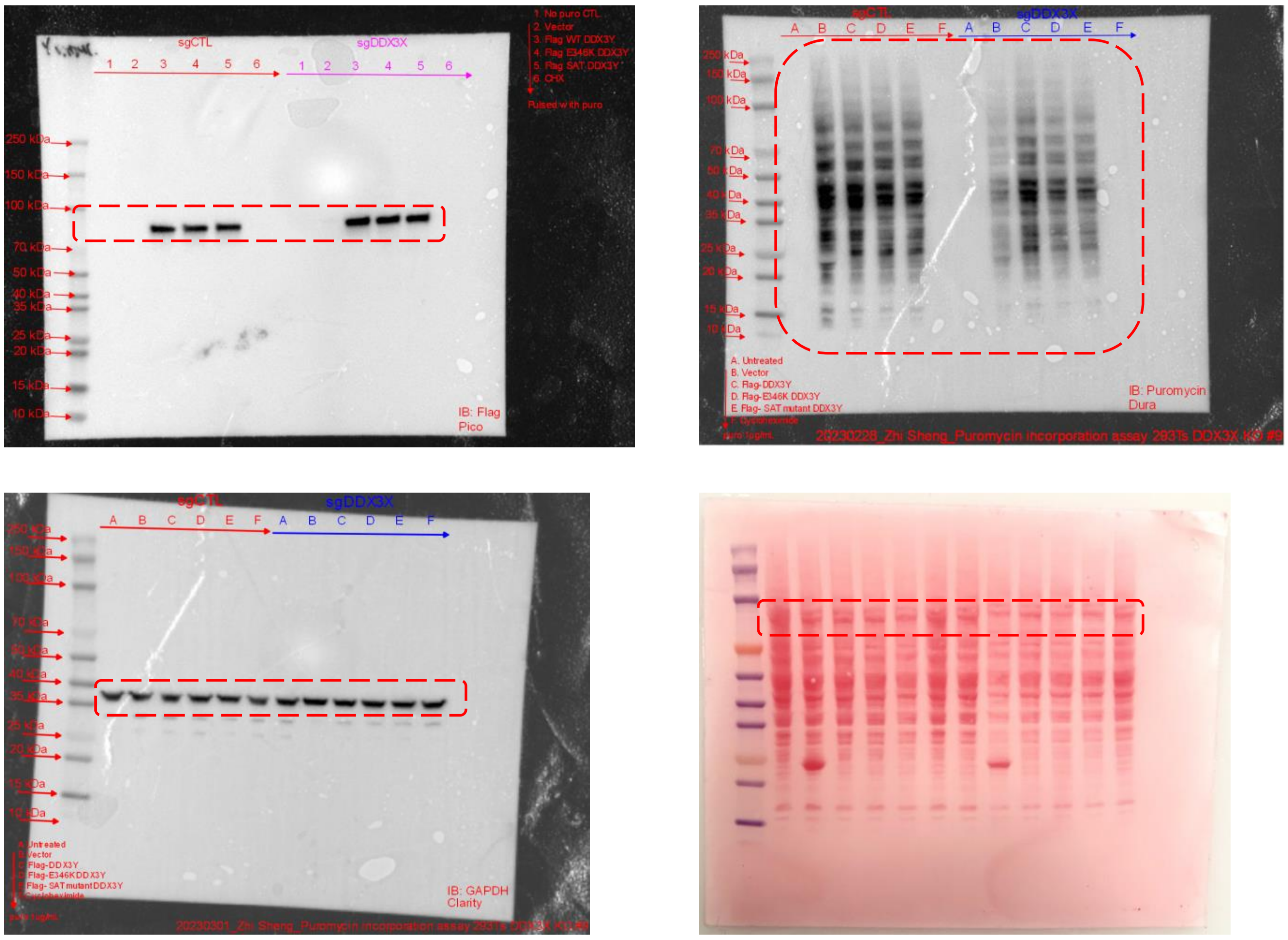

Uncropped blots

Figure S2a

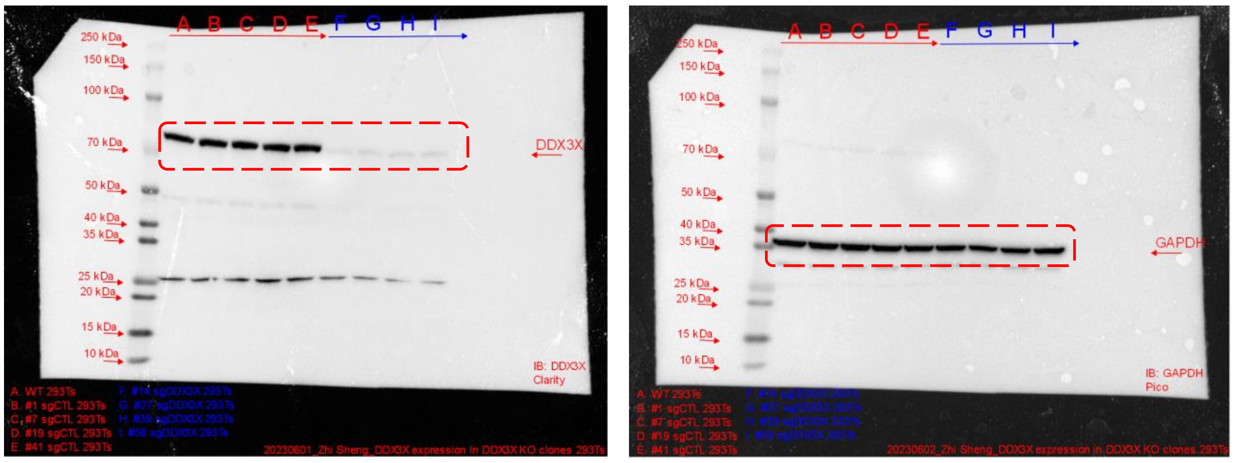

Figure S3a

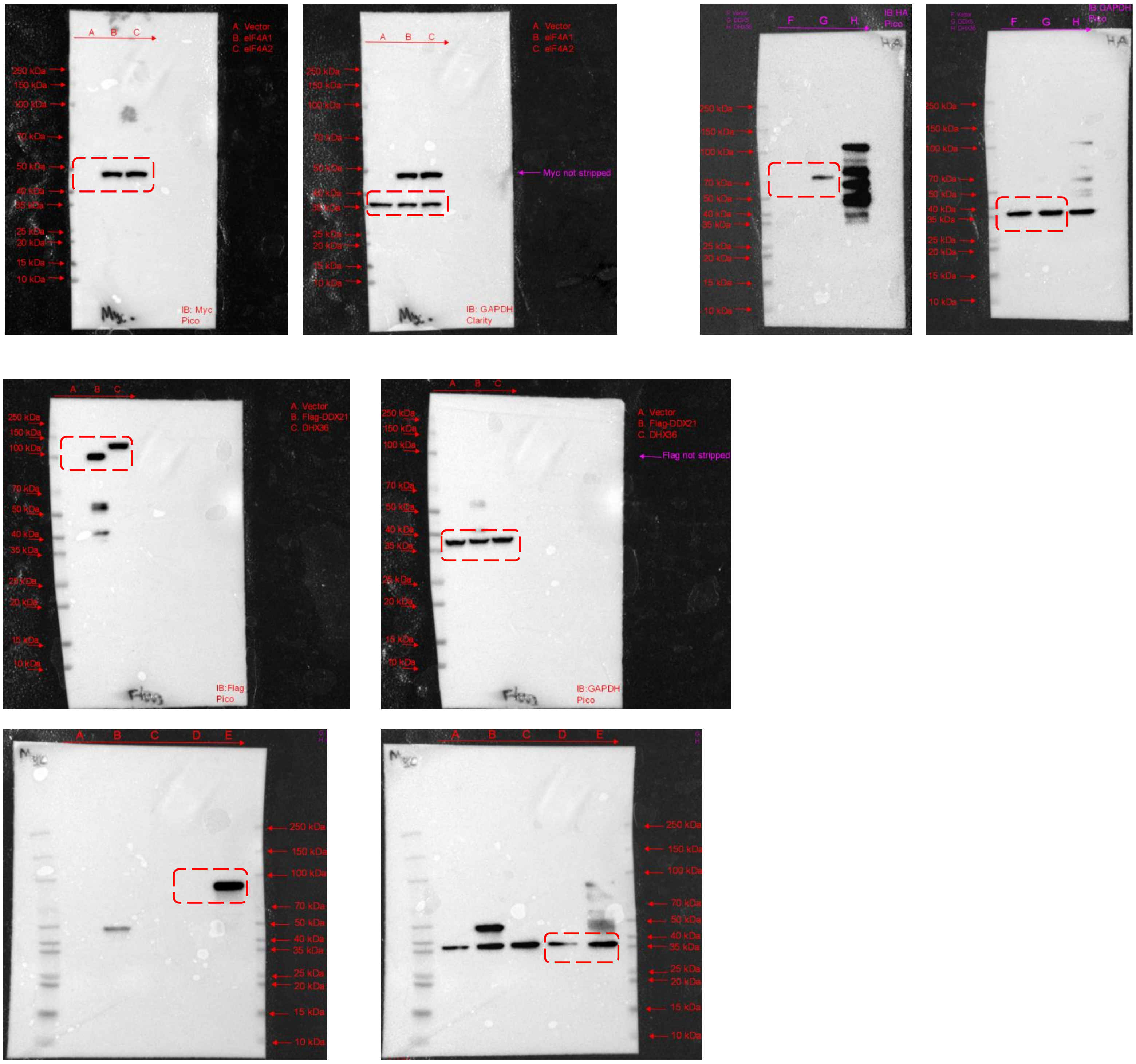

Supplement: Supplementary file 1 — FIGURE S1: (a) Schematic of reporter plasmid in which the complex DvL2 5′UTR was placed upstream of the firefly luciferase (FLuc). The Aequorea coerulescens green fluorescence protein (AcGFP) was placed downstream as an internal control separated by an internal ribosome entry site (IRES). RNA secondary structure was created using ViennaRNA. (b) Gating strategy adopted to determine transfection efficiency within each sample for normalizing bioluminescence signals. (c) The viability of reporter cells (sgCTL and sgDDX3X) treated with various RK‐33 concentrations (0.5, 1, or 2 μM) was determined by an MTS‐based assay normalized against vehicle (DMSO). Staurosporine (STS) was used as a positive kill control. (d) Representative brightfield images of wild‐type (WT) and engineered sgCTL and sgDDX3X 293T cells. (e) Apoptosis/cell death in control and DDX3X knock‐out 293T cells was determined using an Annexin V‐FITC assay kit. Cells were treated with STS as a kill control. Bar chart represents mean ± SEM of three independent experiments. FIGURE S2: (a) Representative immunoblot of WT, sgCTL, and sgDDX3X 293T cells from various clones showing the expression levels of DDX3X and GAPDH (loading control). (b) Firefly luciferase activity of reporter cells, normalized against AcGFP+ levels within each sample before comparing against sgCTL firefly luciferase activity. Cycloheximide was used as a positive control for translational inhibition. (c) Firefly luciferase activity of sgCTL and sgDDX3X reporter cells (Rac1‐FLuc and DvL2‐FLuc) after treatment with increasing concentrations of RK‐33 (0.5, 1, or 2 μM). Data represent mean ± SEM of at least three independent experiments. ns, non‐significant, *p < 0.05; ****p < 0.0001. FIGURE S3: (a) Representative immunoblots of protein samples obtained from sgDDX3X cells transfected with empty vector or plasmids for Myc‐eIF4A1, Myc‐eIF4A2, HA‐DDX5, Flag‐DDX21, Flag‐DHX36, or Myc‐DDX17 and probed for Myc, HA, or Flag. Blots were re‐probed for GAP [file BTM2-10-e10720-s001.pdf]
